# Editorial: Advances on the physiology and cell Biology of invertebrate parasites

**DOI:** 10.3389/fphys.2022.1066273

**Published:** 2022-10-25

**Authors:** Fabio M. Gomes, N. Baranzini, A. Grimaldi, E. J. Lopes-Torres

**Affiliations:** ^1^ Instituto de Biofísica Carlos Chagas Filho, Universidade Federal do Rio de Janeiro, Rio de Janeiro, Brazil; ^2^ University of Insubria, Varese, Italy; ^3^ Laboratório de Helmintologia Romero Lascasas Porto, Universidade do Estado do Rio de Janeiro, Rio de Janeiro, Brazil

**Keywords:** invertebrate parasite, physiology, cell biology, host-parasite dynamics, unionids, stink bugs, tick

This Research Topic aimed to promote the publication of new developments that could deepen our understanding of invertebrate parasitism, such as parasite physiology, reproduction, and microbiota interplay during host-parasite dynamics. Papers published on this topic highlighted the importance of studies on invertebrate parasite cell biology and physiology, and further evaluated the economic impacts caused by these organisms. Advances in molecular biology, biochemistry, and studies on drug discovery used to control invertebrates have not progressed at the same pace of basic research on these complex multicellular organisms (helminths, arthropods, and mollusks). For example, the lack of known specific targets for drugs and pesticides deployed on a large scale worldwide leads to an enhanced exposure of local populations and reduced efficiency of deployed strategies. As a result, economic losses and emerging resistance to treatments have been observed. Crop losses due to pests and diseases are a major threat to the incomes of rural families and food security worldwide (Avelino et al., 2015).

## Interaction between hosts and parasites, and biocontrol

The social behavior of termites is an important obstacle to their biological control, as the dynamic mechanism that coordinates social immunological defense and caste distribution remains undefined. Studies of host-parasite interactions of entomopathogenic fungi in different caste members can improve our understanding of the mechanisms of functional adaptation between pathogen-driven social immunity and termite demographics, evidencing new alternatives for biological controls (Cremer, 2019). Using *Coptotermes formosanus,* Wenhui Zeng et al. studied the impact of demography on the behavior and innate immunity of termites. They observed that infected workers would self-sacrifice to maintain soldier proportion of the group. Indeed, a high proportion of soldiers was demonstrated to be associated with sanitary care of the workers to the nestmate workers and soldiers.

Fruit flies are important vectors of pathogens driving food poisoning in several countries. Parasitoid wasps have been used as biocontrol pests against several pests with varying levels of success. Most studies investigating host-parasitoid models have focused on functional and evolutionary aspects, leaving a gap in knowledge about the physiological mechanisms of tephritid biocontrol agents. Cellular immune responses are central to the host-parasitoid interaction in specific fruit fly and wasp species, presenting greater potential as a biocontrol agent. Rehemah Gwokyalya et al. showed that the cellular immune response of the fruit flies is a key regulator of host-parasitoid dynamics during exposure to the parasitc wasps. *Bactrocera dorsalis* exhibited a stronger cellular immunity, contributing to its successful invasion and establishment across several ecological zones. The authors showed that *D*. *longicaudata* markedly reduced the host immune defenses. This partly explains the high parasitism rates achieved by *D*. *longicaudata*, making it a good control agent for many tephritid fruit fly pests.

## Ultrastructure, omics, and microbiota committed to invertebrate physiology comprehension

The structural plan of unionids - freshwater bivalve mollusks with a parasitic larval stage—is generally conserved, sharing a morphological structure adapted to the parasite’s lifestyle and survival. However, little is known about the neurodevelopment of freshwater mussels (Unionoidea). Viktoria E. Nikishchenko et al. demonstrated that the structures of the sensory, muscle and nervous systems of the larvae of *Nodularia douglasiae* differ from the larval systems of marine bivalve species. The authors found that the glochidia sensory system included four pairs of tubulin-lir multicilia hair cells and non-ciliar tubulin-lir cells. These cells synthesize the neuropeptide FMRFamide and were identified as afferent neurons collecting information from peripheral tubulin-lir hair sensory cells to nervous regulators. The authors concluded that the nervous system of *N*. *douglasiae* glochidia is drastically different from other mollusks and lophotrochozoans because of the absence of an apical organ and the location and composition of FMRFamide and 5-HT cells.

Stink bugs are the major pests of lychee trees. These insects’ secretions are not only harmful to plants, but also to humans. Omics data are useful for understanding the growth and development of insects and identifying pest control targets (Wu et al., 2017). Understanding the growth and development of lychee bugs, their choice of hosts, digestion and food detoxification, and their reproductive behavior provides essential baseline information for novel target genes that can be used to design new control strategies. Lin Cheng et al. described a total of 462 unigenes of *Tessaratoma papillosa* related to growth and development. 1,851 unigenes related to digestion and detoxification, and 70 unigenes related to olfaction. They showed that the *T*. *papillosa* major life activity genes are uniformly expressed across all developmental stages. However, adult midgut gene expression was utterly different from that of the nymphs. Similarly, female fat body genes exhibited distinct expression patterns compared to that of males and nymphs, providing reference information for putative genes targeting the control of this herbivorous insect.

The Brown dog tick is the most widespread tick in the world and is a predominant vector of multiple pathogens affecting the wildlife, domestic animals, and humans. The One Health paradigm emphasizes the importance of studying invertebrate physiology, especially in parasitic arthropods with non-specific relationships with their vertebrate hosts (euryxene). Tick gut microbiota is influenced by several factors, including tick species, dietary blood supply, and physiological stress. Nevertheless, the composition of tick microbiota has remained largely understudied, especially when compared to insect’s. Liping An et al. found that the microbial community composition of *Rhipicephalus sanguineus* changed significantly with starvation stress. The bacteria *Coxiella* spp. of *R*. *sanguineus* gradually decreased thorough the starvation. Nymphs were treated with ofloxacin and allowed to develop into adults. Adult blood-sucking rate, adult weight after a blood meal, fecundity, and feeding period of the newly hatched larvae were all affected to varying degrees. The control of microbial composition or diversity through antibiotics can have an impact on tick development and affect it to varying degrees.

Overall, these articles explored different tools and techniques, from a wide range of experts that strongly contributed toward the advance in the physiology of invertebrate parasites. Advanced research in cell biology and a better understanding of invertebrate physiology associated with economic losses are essential to assess the effectiveness of parasite and pest protection strategies, enabling sustainable interventions with biological or pharmacological controls (Cerda et al., 2017).
